# How Personality Relates to Distress in Parents during the Covid-19 Lockdown: The Mediating Role of Child’s Emotional and Behavioral Difficulties and the Moderating Effect of Living with Other People

**DOI:** 10.3390/ijerph17176236

**Published:** 2020-08-27

**Authors:** Cristina Mazza, Eleonora Ricci, Daniela Marchetti, Lilybeth Fontanesi, Serena Di Giandomenico, Maria Cristina Verrocchio, Paolo Roma

**Affiliations:** 1 Department of Neuroscience, Imaging and Clinical Sciences, G. d’Annunzio University of Chieti-Pescara, 66100 Chieti (CH), Italy; mazzacristina87@gmail.com; 2 Department of Human Neuroscience, Sapienza University of Rome, 00185 Rome (RM), Italy; eleonoraricci25@gmail.com (E.R.); paolo.roma@uniroma1.it (P.R.); 3 Department of Psychological, Health and Territorial Sciences, G. d’Annunzio University of Chieti-Pescara, 66100 Chieti (CH), Italy; d.marchetti@unich.it (D.M.); lilybeth.fontanesi@gmail.com (L.F.); serenadigiandomenico@libero.it (S.D.G.)

**Keywords:** mental health, distress, neuroticism, emotional stability, hyperactivity–inattention, BIF-10, SDQ-P, GHQ-12, parenting

## Abstract

Since the initiation of the COVID-19 lockdown, Italian parents have been forced to manage their children at home. The present study aimed at investigating the psychological distress of parents during the lockdown, identifying contributing factors. An online survey was administered to 833 participants from 3 to 15 April 2020. Mediation and moderated mediation models were run to explore the association between parent neuroticism and parent distress, mediated by child hyperactivity–inattention and child emotional symptoms, and the moderating effect of living only with child(ren) on the direct and indirect effects of parent neuroticism on parent distress. For parents living only with child(ren), high levels of psychological distress depended exclusively on their levels of neuroticism. For parents living with at least one other person in addition to child(ren), distress levels were also mediated by child behavioral and emotional difficulties. Motherhood emerged as a significant factor contributing to greater distress. Furthermore, parent psychological distress decreased in line with increased child age. The results confirm that neuroticism is an important risk factor for mental health. Preventive measures should be primarily target multicomponent families with younger children and directed towards parents who are already known to present emotional instability and to parents of children who have received local mental health assistance for behavioral and/or emotional difficulties.

## 1. Introduction

Following the uncontrolled and exceptional spread of COVID-19, the Italian government imposed a lockdown on the entire country on 9 March 2020. Similar to lockdowns in other countries, the Italian lockdown has exposed parents to a long and unexpected period of managing their children’s care and schooling at home, while also—for many—continuing to work remotely [1,2]. A recent review of the literature [3] highlighted that lengthy lockdowns may have a significant negative impact on mental health and well-being. In particular, studies have reported that a long duration of quarantine may increase psychological distress [4], depressive symptoms, stress, and anxiety [5,6,7]. A recent study [8] assessed the psychological impact of the COVID-19 lockdown in 2766 subjects from all regions of Italy, showing a high prevalence of psychological symptomatology. The findings underlined that female gender and the personality domains of negative affect and detachment were associated with higher levels of depression, anxiety, and stress. Furthermore, knowing someone who had been infected with COVID-19 was associated with increased levels of depression and stress, and a history of stressful situations and medical problems was associated with higher levels of depression and anxiety. Finally, persons with a family member who had been infected and young persons who were continuing to work outside the home reported higher levels of anxiety and stress, respectively.

Studies on the psychological effects of quarantine and lockdown measures during previous epidemics and pandemics (e.g., SARS, H1N1, Ebola, MERS, equine influenza) have shown that 30% of children and 25% of parents show high levels of psychological distress [9]. Furthermore, other research [10,11] has underlined that being a parent contributes to greater psychological distress during a lockdown or quarantine and that living with children is significantly associated with increased concern for personal and family health.

To the best of our knowledge, no prior study has investigated the mental health of parents during the COVID-19 lockdown. Nonetheless, recent commentaries [1,2] have highlighted the urgency of assessing the mental health of parents who, following the closure of schools, are forced to care for their children at home whilst also—in many cases—working remotely. In order to assess the mental health of parents during the present lockdown, we gave due consideration to the association between psychological distress and personality traits. The literature in this area shows that some personality features contribute to sensitive parenting, whereas other features are linked to less responsive parenting [12]. In particular, the dimension of *neuroticism* (also referred to as *emotional stability*) has received significant empirical attention. Studies have shown that parents with high neuroticism tend to show high psychological distress and anxiety, and to experience negative life events more severely [13,14]. Research on neuroticism has largely focused on mothers with depressive symptoms, highlighting that highly neurotic mothers tend to be less warm and sensitive [15,16,17,18] and more focused on themselves and their own distress, rather than their children’s needs [13,14].

Parental neuroticism has been shown to be associated with increased child mental health problems. Specifically, previous studies have found associations between parental neuroticism and abnormal emotional symptoms in younger children, conduct disorders in older children, and hyperactivity in children of all ages [19]. It is well known that families of children with disabilities or behavioral and emotional problems constantly struggle with the difficulties associated with managing these children’s issues. Recent studies [20,21,22] have shown that low socioeconomic status, a high burden of care, and a lack of psychological support can contribute to greater psychological distress among families. Indeed, high levels of psychological distress have been recorded among families of children with disabilities or behavioral and emotional problems [23], with the parents reporting high levels of stress and depression and difficult emotions, including anger, grief, guilt, and inadequacy [24,25]. Overall, neuroticism was found to predict child behavioral problems, both independently and as a mediator linked to parental depression. In turn, neuroticism was found to be significantly related to parental emotional response to children’s emotional and behavioral difficulties. Thus, parents with higher neuroticism may have reacted to children’s problems and needs with more negative emotionality and reported a higher level of psychological distress. Thus, more neurotic parents (especially mothers) experienced more malaise, had a less happy relationship with their child, which in turn expressed more behavioral problems. This in turn may have exaggerated their anxiety, depression and moodiness. Hence the concept of a vicious cycle [26].

The results of prior studies have also shown that the perceived social support was negatively associated with psychological distress among parents. Social support, indeed, may improve personal control (i.e., individuals’ beliefs regarding their ability to control their life situations), and in turn, improve mental health [27,28]. In line with this finding, O’leimat et al. [29] recently found that divorced parents of children with psychiatric disorder had significantly higher psychological distress than married parents: this outcome may be due to the fact that divorced parents usually have no psychological support from their partner, which might increase their distress. Given the lockdown suddenly imposed by the Italian Government, in the present study “people living with” was considered as a proxy variable to the social support, independently from the marital status or the kinship ties.

Starting from this evidence, the present study set out to test the following hypotheses:

**Hypothesis** **1:** Neuroticism would be positively related to distress in parents, even after controlling for parent age and parental role, income, and number of children, as well as child age. Higher levels of parent neuroticism would increase parent psychological distress during the lockdown.

**Hypothesis** **2:** Child hyperactivity–inattention and child emotional symptoms would mediate the relationship between parent neuroticism and parent psychological distress during the lockdown (simple mediational model), even after controlling for parent age and parental role, income, and number of children, as well as child age. We expected that higher levels of parent neuroticism would increase parent psychological distress during the lockdown, via greater child hyperactivity–inattention (2a) and more child emotional symptoms (2b).

**Hypothesis** **3:** Living only with child(ren) would moderate the positive relationship between parent neuroticism and parent psychological distress during the lockdown (3a), as well as the positive relationship between child hyperactivity–inattention (3b), child emotional symptoms (3c), and parent psychological distress during the lockdown (moderated mediation models). All these relations would be stronger for parents living only with child(ren).

**Figure 1:**
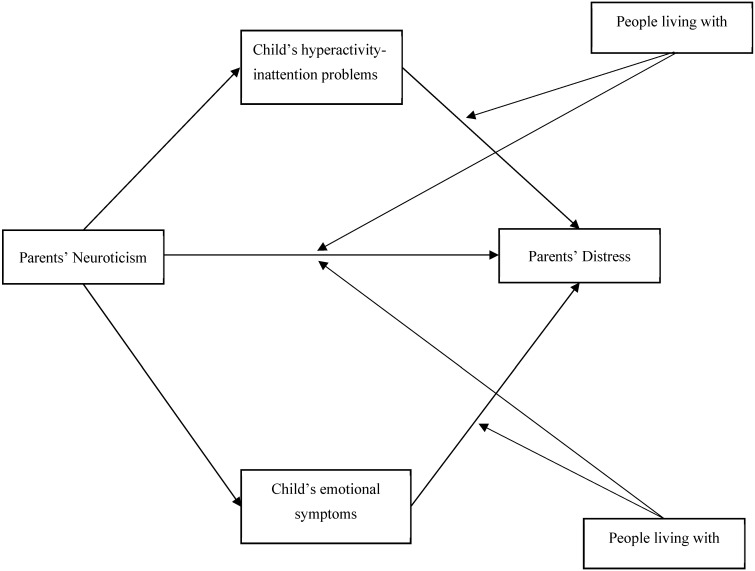
Proposed moderated mediation model.

Figure 1 shows the proposed moderated mediation model, in which both mediators were considered simultaneously.

## 2. Material and Methods

### 2.1. Procedures

A Qualtrics online survey was administered from 3 to 15 April 2020, 1 month after the initiation of the COVID-19 lockdown in Italy, which participants accessed via a designated link. The link was disseminated through the main means of communication and social networks, in order to reach a large number of parents, both mothers and fathers, indiscriminately. All participants voluntarily responded to the anonymous questionnaire and indicated their informed consent within. They did not receive any compensation for their participation, but it was specified that the results of the research would be made available upon request. Parents were asked to answer truthfully and referring to their own experience, in order to analyze how they were facing the challenges arising from the moment of health emergency they were experiencing in order to be able to promptly implement adequate support interventions. The procedures were clearly explained, and participants could interrupt or quit the survey at any point without explaining their reasons. The study was approved by the local ethics committee (Board of the Department of Human Neuroscience, Faculty of Medicine and Dentistry, Sapienza University of Rome).

### 2.2. Participants

A total of 1180 parents participated in the survey. Inclusion criteria were: (a) parent aged 18 years or older, with (b) at least one child aged 3–13 years living with them during the lockdown. Participants with an incomplete questionnaire were excluded from the analyses (*N* = 247), as well as those who had a child under 3 years (*N* = 37) or over 13 years (*N* = 63). Hence, the final sample consisted of 833 parents: 736 mothers (88.4%) and 97 fathers (11.6%), aged 23–67 years (*M* = 40.61, *SD* = 6.3), living with one to six children (*M* = 1.83, *SD* = 0.75) aged 3–13 years (*M* = 7.57, *SD* = 3.2). Most of the sample (*N* = 325, 39%) had a high school diploma and were employed (*N* = 704, 84.5%). Of the total sample of parents, 38.1% reported that they were continuing to work remotely from home throughout the lockdown (*N* = 317), and 84.5% were spending the lockdown at home with their children as well as at least one other person (*N* = 704). More descriptive statistics, including all of the characteristics considered, are presented in Table 1.

### 2.3. Measures

In addition to a questionnaire designed to investigate demographic variables and factors related to the COVID-19 lockdown, the following measures were employed.

The 10-item Big Five Inventory (BFI-10) [30,31] was used to measure personality features. This is a 10-item scale designed to assess the Big Five dimensions (i.e., openness to experience, agreeableness, extroversion, emotional stability/neuroticism, conscientiousness). Each dimension is rated on a 5-point Likert scale ranging from 1 (*totally disagree*) to 5 (*totally agree*). On the basis of the literature, in the present study, we assessed the Emotional Stability/Neuroticism dimension (i.e., items 4 and 9) and its association with parent distress. It is worth noting that Emotional Stability dimension is defined as the opposite of Neuroticism (i.e., the tendency to experience negative affect). In our sample, internal consistency on the Emotional Stability/Neuroticism subscale was assessed using the Spearman–Brown coefficient (*r_s_* = 0.46), which performs better than Cronbach’s alpha in assessing the internal consistency of two-item subscales [32]. In the Italian validation of the BFI-10 [33], Spearman-Brown coefficients were found to be 0.50 or higher and thus considered acceptable, since each subscale consists of only two items [34]. 

The Strengths and Difficulties Questionnaire (SDQ) [35] was employed to assess hyperactivity–inattention and emotional symptoms among children. The SDQ is a multi-informant instrument for screening developmental psychopathology. Each subscale (Conduct Problems, Hyperactivity–inattention, Emotional Symptoms, Peer Problems) contains five items rated on a 3-point Likert scale ranging from 0 (*not true*) to 2 (*certainly true*). In the present study, the Hyperactivity–inattention and Emotional Symptoms subscales were used. These subscales were shown to have good internal consistency, with Cronbach’s alphas of 0.89 and 0.73, respectively [36]. In our sample, the subscales had internal consistency values of 0.76 and 0.70, respectively.

The General Health Questionnaire-12 (GHQ-12) [37] was adopted to detect minor psychiatric disorders. The GHQ-12 is a self-administered questionnaire to assess mental illnesses of a non-psychotic nature. It consists of 12 items measured on a 4-point Likert scale ranging from 1 (*less than usual*) to 4 (*much more than usual*). The GHQ-12 has been shown to be a reliable instrument, as indicated by a Cronbach’s alpha of 0.84 [38,39,40]. In our sample, the GHQ-12 obtained high reliability, with a Cronbach’s alpha of 0.84. 

## 3. Statistical Analysis 

The mediation and moderated mediation models were run using PROCESS Version 2 [41], as developed by Preacher and Hayes [42] for SPSS, version 25 (IBM, Armonk, NY, USA). Moderated mediation tests simple mediation models (i.e., to determine whether a given variable or mediator accounts for some or all of the relationship between two other variables) that may differ according to additional variables (e.g., when the mediation pathway is only present for individuals with higher/present or lower/absent levels of certain variables). PROCESS estimates indirect effects (i.e., mediation) and conditional indirect effects (i.e., moderated mediation) using bootstrap confidence intervals. In the present study, the bias-corrected 95% confidence interval (CI) was calculated with 5000 bootstrapping resamples. Effects were considered significant when the resulting confidence interval did not contain 0. All measures were treated as continuous variables except for the ordinal covariate “income” and the dummy covariate “living only with child(ren).” With respect to PROCESS Model Templates [41], we first tested a simple mediation model (Model 4) to explore whether the association between parent neuroticism and parent distress was mediated by child hyperactivity–inattention and child emotional symptoms. Next, we tested Model 15 to verify the moderated effect of living only with child(ren) on the direct and indirect effects of neuroticism on parent distress. Both models were tested after controlling for parent age and parental role, income, and number of children, as well as child age.

## 4. Results

Correlations among the variables are reported in Table 2. All significant correlations were in the expected directions. Of note, higher scores of BFI-10 Emotional Stability corresponded to lower levels of Neuroticism. Neuroticism was positively related to the outcome (*r_GHQ_* = −0.318) and to both mediators (*r_SDQ H-I_* = −0.195; *r_SDQ ES_* = −0.144), but not to living only with child(ren) (*r_pb_* = 0.003). The SDQ Hyperactivity–Inattention subscale was positively correlated with parent distress (*r_GHQ_* = 0.201) and not correlated with living only with child(ren) (*r_pb_* = 0.041). The same was true for the SDQ Emotional Symptoms subscale, which was positively correlated with parent distress (*r_GHQ_* = 0.259) and not correlated with living only with child(ren) (*r_pb_* = −0.017).

### 4.1. Mediation Test

We next tested the prediction concerning the link between parent neuroticism and parent distress and the prediction concerning the mediating role of child hyperactivity–inattention and child emotional symptoms. The total effect of neuroticism on distress (*B* = −0.899 [*SE* = 0.10] *p* < 0.001 [CI = −1.089, −0.709]) was significant. Furthermore, as shown in Table 3, the indirect effect of neuroticism on distress via child hyperactivity–inattention and child emotional symptoms was negative (−0.120), and the bootstrapped 95% CI did not include 0 [−0.182, −0.63]. 

Furthermore, the covariates “child age” and “parental role” showed a significant negative association with parent distress. The final mediation model explained 17% of the variance in parent distress. The direct effect of neuroticism explained 87.4% of the total effect on parent distress, whereas the indirect effects of the mediators were 4.6% and 8% for child hyperactivity–inattention and child emotional symptoms, respectively. Thus, Hypotheses 1 and 2 (a, b) were supported. 

### 4.2. Moderated Mediation Test

**Figure 2:**
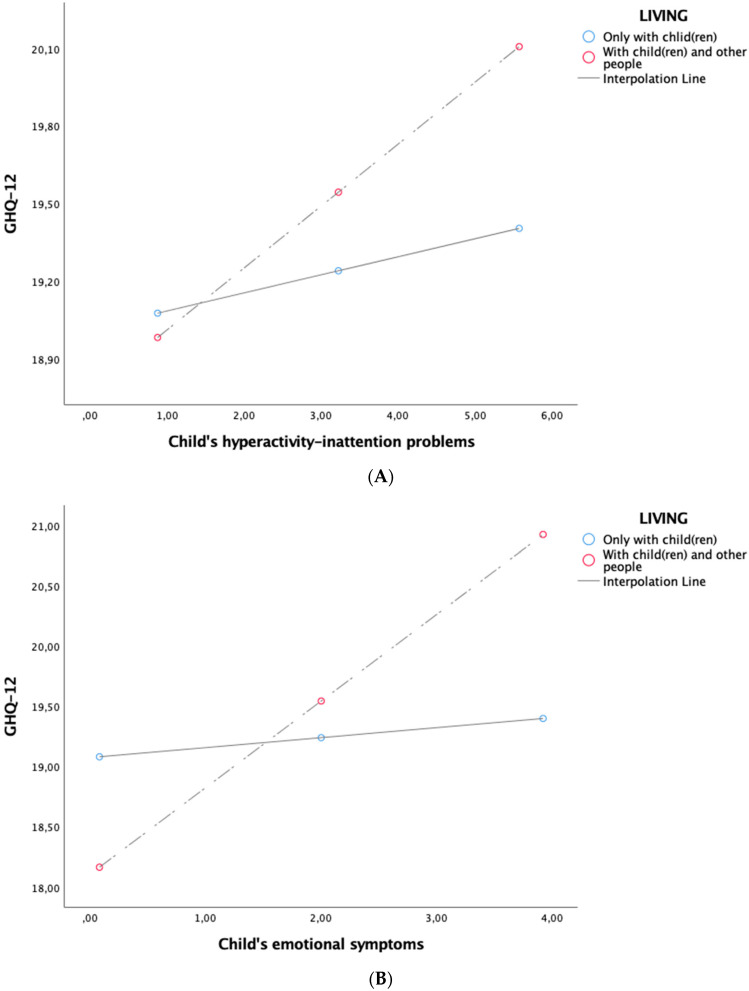
Simple slope analyses (**A**,**B**).

Table 4 presents the results of the mediation analyses relating to the conditional direct and indirect effects. Hypothesis 3 further predicted that the strength of the direct (3a) and indirect effects of parent neuroticism on parent distress through child hyperactivity–inattention (3b) and child emotional symptoms (3c) would be conditional on living only with child(ren). The direct effect of parent neuroticism on parent distress was stronger for parents living only with child(ren) (β = −1.234 (*SE* = 0.23), 95% CI (−1.692, −0.775)), compared to those living with at least one other person (β = −0.665 (*SE* = 0.11), 95% CI (−0.871, −0.460)). Thus, hypothesis 3a was confirmed. The indirect effect of parent neuroticism on parent distress via child hyperactivity–inattention was significant only for parents living with at least one other person (β = −0.049 (*SE* = 0.02), 95% CI (−0.100, −0.005)). In contrast, for parents living only with child(ren), the indirect effect of parent neuroticism on parent distress via child hyperactivity–inattention was not significant (β = −0.014 (*SE* = 0.05), 95% CI (−0.109, 0.068)). The same pattern was found for the indirect effect of parent neuroticism on parent distress via child emotional symptoms: This was significant only for parents living with at least one other person (β = −0.093 (*SE* = 0.03), 95% CI (−0.151, −0.044)). In contrast, for parents living only with child(ren), the indirect effect of parent neuroticism on parent distress via child emotional symptoms was not significant (β = −0.011 (*SE* = 0.03), 95% CI (−0.069, 0.055)). Once again, the covariates “child age” and “parental role” showed significant negative associations with parent distress. Taken together, these results revealed that the indirect effect of parent neuroticism on parent distress via child hyperactivity–inattention and child emotional symptoms was significant only for parents living with child(ren) and at least one other person, disconfirming Hypotheses 3b and 3c. Figure 2 showed the simple slope analyses of the effect of living only with child(ren) on the relationship between parent distress and child hyperactivity–inattention (A) and emotional symptoms (B).

## 5. Discussion

The results of the present study highlighted that there is a positive relation between parent neuroticism and parent distress, after controlling for parent age, income, and number of children. Indeed, in support of our first hypothesis, high levels of parent neuroticism—characterized by patterns of worrying, nervousness, emotional instability, and feelings of inadequacy—have been found to increase parent psychological distress, mainly in terms of anxiety, depression, and social dysfunction [43]. In other words, parents who report high neuroticism may experience a wide array of negative emotions (e.g., anger, frustration) and cognitions (e.g., pessimism, perfectionism) and be more prone to developing non-specific psychological distress [44], anxiety and depression, supporting the results of Kotov, Gamez, Schmidt, and Watson’s [45] extensive meta-analysis and other longitudinal studies [46]. Neuroticism, indeed, is conceptualized as the trait that reflects individual differences in the experience and expression of negative emotion. It has also been defined in terms of its capacity to amplify (i.e., interact with) the negatively valenced emotional effects of stressors: For these reasons this trait has often been hypothesized to be a vulnerability for depression and other internalizing disorders [47]. Furthermore, the present findings support previous studies showing that parents with high levels of neuroticism tend to experience negative life events more severely [13,14].

It is well known that neuroticism makes parents more focused on themselves, less warm and sensitive towards their children, and less focused on their children’s needs [15,16,17,18]. Parental neuroticism has also been shown to be associated with increased child psychological problems [19]. Indeed, the children of neurotic parents tend to develop abnormal emotional symptoms, conduct disorders, and hyperactivity. Parental psychological distress has also been recorded among families of children with behavioral problems [23], with parents reporting high levels of stress and depression and negative emotions, including anger, grief, guilt, and inadequacy in their ability to manage their children’s difficulties [24,25]. Accordingly, our findings showed that child hyperactivity–inattention and child emotional symptoms increased psychological distress among parents during the lockdown, triggering a self-perpetuating cycle of the negative effects of neuroticism, confirming our second hypothesis. 

With regards to our third hypothesis, interesting results emerged. Specifically, for parents living only with child(ren), the effect of neuroticism on their psychological distress was greater—almost double—than that experienced by parents living with at least one other person. In contrast, for parents living with at least one other person, the indirect effect of child hyperactivity–inattention and child emotional symptoms contributed to greater psychological distress, in association with their levels of neuroticism. In other words, while parents living only with child(ren) experienced high psychological distress as an exclusive function of their neuroticism, parents living with at least one other person had their distress mediated by their child’s behavioral and emotional difficulties. A possible explanation for this result, which ran contrary to our expectations, is that neurotic parents may have felt judged as inadequate and incompetent by the person(s) in their household when their children showed emotional and/or behavioral problems. Furthermore, conflicts between household members may have emerged in response to their management of their children’s emotional and/or behavioral problems. 

Generally, our results showed that motherhood emerged as a significant factor contributing to greater distress. This finding, in line with previous studies, could be explained by two simultaneous factors: (a) the association between the female gender and increased psychological distress and internalizing symptoms and (b) the sociocultural relation of the maternal role to childcare [26,48,49,50]. Furthermore, when child age increased, parent psychological distress decreased. This may have been due to parents’ limited parenting competence and experience when children were young. Indeed, previous studies have shown that, as children age and become increasingly self-reliant, parents gain parenting knowledge and experience less parenting stress [51,52].

## 6. Conclusions

Overall, our findings confirm the importance of personality traits in psychological well-being, showing that neuroticism is an important risk factor for poor mental health, especially in the context of stressful situations, such as the COVID-19 outbreak. Support interventions should be immediately activated by mental health and social services for parents who are already known to exhibit emotional instability (e.g., parents who have confirmed this trait during child custody disputes), in order to prevent chronic and amplified negative manifestations, which may have a knock-on effect on children’s psychological health and a predictable return loop. Preventive measures should also be directed towards parents of children who have already received local mental health assistance for behavioral and/or emotional difficulties. These measures should primarily target multicomponent families with younger child(ren). Finally, although more difficult to implement, broad spectrum assessment should be undertaken to detect parents with a high level of neuroticism, in order to develop primary prevention actions [53]. 

The present study, with a large sample size and validated psychological measures employed, provides insight into the association between parent personality traits, parent distress, and child behavioral and emotional problems from an intergenerational perspective, in the context of great stress (e.g., the COVID-19 pandemic). Above all, it contributes to our understanding of the role of the household members, unexpectedly unsupportive with parents when there are children who exhibit emotional and behavioral difficulties. This finding could lead future research to investigate the role of other people in the household and assess the quality of the relationship between household members. Future study efforts could also explore the combined influence of parent psychological distress and parenting styles in the household, both on children wellbeing and attachment styles.

The main limitation of the research is that the sample was mainly composed of mothers, which reduces the generalizability of the findings to fathers. With respect to this latter limitation, the gender imbalance in the present study has been consistently demonstrated, reported, and addressed in previous research [54,55]. Furthermore, child behavioral and emotional functioning was not directly rated but rated by parents. 

## Figures and Tables

**Table 1:** Descriptive statistics of parents.

Characteristic	Group	*N* (%)
Parental role		
	Mother	736 (88.4)
	Father	97 (11.6)
Educational level		
	Primary school diploma	3 (0.4)
	Middle school diploma	44 (5.3)
	High school diploma	325 (39)
	Graduate	274 (32.9)
	Postgraduate	187 (22.4)
Occupation		
	Unemployed	129 (15.5)
	Employed	704 (84.5)
Income		
	Low (0–15,000)	99 (11.9)
	Medium–low (16,000–33,000)	377 (45.3)
	Medium–high (34,000–55,000)	273 (32.8)
	High (over 55,000)	84 (10.1)
Citizenship		
	Italian	818 (98.2)
	Foreign	15 (1.8)
Marital status		
	Unmarried	26 (3.1)
	Married	614 (73.7)
	Separated/Divorced	68 (8.2)
	Widower	4 (0.5)
	Cohabitant	121 (14.5)
Living with		
	Only child(ren)	129 (15.5)
	Child(ren) and others	704 (84.5)
Condition (home/work)		
	Must go to work	127 (15.2)
	Working from home	317 (38.1)
	Can stay home/work activity stopped	389 (47.6)

**Table 2:** Descriptive statistics and intercorrelations.

Dimension	M	SD	1	2	3	4	5
1. GHQ-12	19.50	5.9	-				
2. BFI-10 Emotional Stability/Neuroticism	6.34	2.0	−0.318 **	-			
3. SDQ HYP	3.23	2.4	0.201 **	−0.195 **	-		
4. SDQ ES	2.00	1.9	0.259 **	−0.144 **	0.298 **	-	
5. Living only with child(ren) ^	-	-	0.027	0.003	0.041	0.017	-

Notes. GHQ-12 = General Health Questionnaire-12; HYP = child hyperactivity–inattention; ES = child emotional symptoms. *p* < 0.01 **. ^ Point-biserial correlations were computed.

**Table 3:** Simple mediation results (*N* = 833).

Predictors	HYP		ES		GHQ-12		
	β (SE)	*p*	β (SE)	*p*	β (SE)		*p*
**Independent variables**							
Neuroticism	−0.205 (0.04)	<0.001	−0.129 (0.03)	<0.001	−0.779 (0.10)		<0.001
HYP	-	-	-	-	0.206 (0.09)		0.017
ES	-	-	-	-	0.600 (0.10)		<0.001
**Covariates**							
Parent age	0.015 (0.02)	0.358	−0.007 (0.01)	0.577	0.055 (0.04)		0.150
Parental role (*ref. motherhood*)	−0.297 (0.03)	0.245	−0.453 (0.21)	0.032	−1.463 (0.60)		0.015
Income	−0.259 (0.10)	0.010	−0.193 (0.08)	0.021	0.231 (0.24)		0.332
Child age	−0.108 (0.03)	0.001	0.055 (0.03)	0.035	−0.177 (0.08)		0.018
N° children	0.077 (0.11)	0.486	0.071 (0.09)	0.439	0.471 (0.26)		0.072
**R^2^**	0.06		0.04		0.17		
**F (df)**	9.447 (6826) ***		6.073 (6826) ***		20.965 (8824) ***		
**Bootstrap indirect effects on GHQ-12**		**GHQ-12**			**95% CI**		
		β (SE)			LL		UL
Total		−0.120 (0.03)			−0.182		−0.063
HYP		−0.042 (0.02)			−0.087		−0.004
ES		−0.077 (0.02)			−0.127		−0.034

Notes. HYP = child hyperactivity–inattention; ES = child emotional symptoms; GHQ-12 = General Health Questionnaire-12. Bootstrap sample size = 5000 (two-tailed); *p* < 0.001 ***.

**Table 4:** Moderated mediation results (*N* = 833).

Predictor	β(SE)	*p*	95% CI	
			LL	UL
Neuroticism	−1.234 (0.23)	<0.001	−1.692	−0.775
HYP	0.070 (0.21)	0.741	−0.343	0.483
ES	0.083 (0.24)	0.619	−0.393	0.559
Living only with child(ren)	−5.118 (1.9)	0.006	−8.830	−1.406
Neuroticism × Living only with child(ren)	0.568 (0.26)	0.021	0.067	1.070
HYP × Living only with child(ren)	0.170 (0.23)	0.459	−0.280	0.620
ES × Living only with child(ren)	0.636 (0.27)	0.017	0.113	1.159
Parent age	0.058 (0.04)	0.131	−0.017	0.132
Parental role (*ref. motherhood*)	−1.468 (0.60)	0.015	−2.647	−0.288
Income	0.178 (0.24)	0.460	−0.295	0.651
Child age	−0.165 (0.08)	0.028	−0.312	−0.018
N° children	0.468 (0.26)	0.073	−0.043	0.979
R^2^	0.18			
F	15.138 (12820) ***			

Notes. HYP = child hyperactivity–inattention; ES = child emotional symptoms; GHQ-12 = General Health Questionnaire-12. Bootstrap sample size = 5000 (two-tailed); *p* < 0.001 ***.
